# The psychometric validation of a US English satisfaction measure for patients with benign prostatic hyperplasia and lower urinary tract symptoms

**DOI:** 10.1186/1477-7525-7-55

**Published:** 2009-06-19

**Authors:** Libby Black, Alyson Grove, Betsy Morrill

**Affiliations:** 1 GlaxoSmithKline, Research Triangle Park, NC, USA; 2 Roundpeg Research, Abingdon, Oxon, UK

## Abstract

**Background:**

The purpose of the current study was to validate the US English Patient Perception of Study Medication (PPSM) questionnaire, which measures patient satisfaction with Benign Prostatic Hyperplasia (BPH) treatment and was administered to men with BPH lower urinary tract symptoms (LUTS) enrolled in a multi-national clinical trial.

**Methods:**

Patients with moderate to severe BPH symptoms completed three disease-specific measures: The International Prostate Symptom Score (IPSS), the BPH Impact Index (BII) and the PPSM, at baseline (after completion of the placebo run-in period) and at every 13-week clinic visit thereafter for the duration of the study treatment period. The PPSM was analysed to assess its variability, reliability and validity.

**Results:**

There were 879 patients included in the analyses, with a mean age of 66.7 years. The PPSM was found to comprise two factors – PPSM-Global and PPSM-Pain, with a Total Score ranging from 7 to 49. It demonstrated good internal consistency (Cronbach's alpha ranged from .95 to .97) and also demonstrated convergent validity through significant correlations with the IPSS (.48 to .58), IPSS Quality of Life (QoL) item (.41 to .63) and BII (.31 to .45) and known-groups validity against the IPSS, IPSS QoL item and BII.

**Conclusion:**

Results support the use of the PPSM as a measure of satisfaction in BPH patient groups.

## Background

Benign Prostatic Hyperplasia (BPH) is the most common benign neoplasm in the ageing male population with pathological changes found in 88% of men in their ninth decade and symptoms reported in nearly 50% of men aged ≥ 50 years in the general population [1]. The known proximal cause of BPH is age-related prostate growth that is stimulated primarily by the presence of dihydrotestosterone (DHT). DHT is formed when testosterone is reduced through the activity of the 5 α-reductase enzymes type 1 and type 2, although type 2 is considered primarily responsible for this conversion in the prostate. Prostatic growth may lead to urethral obstruction which causes lower urinary tract symptoms (LUTS) such as urge, frequency, nocturia and incontinence that interfere with normal activities.

BPH with LUTS is a chronic condition, which is potentially progressive. This progression includes an increase in prostate volume, deterioration in LUTS and maximum urinary flow rate (Q_MAX_), increased risk of acute urinary retention (AUR) and BPH-related surgery and a deterioration of BPH-related quality of life [2,3] 5α-reductase inhibitors (5ARIs) have been shown to interrupt disease progression in patients with BPH by impeding the conversion of testosterone to dihydrotestosterone, which is believed to cause hyperplastic growth of the prostate, and can also reduce prostate volume for patients with enlarged prostates [2,4].

In a pooled analysis of three placebo-controlled, 2-year double-blind clinical trials, Roehrborn et al [4] examined the efficacy and safety of dutasteride, a potent type 1 and type 2 5ARI. Dutasteride was shown to be associated with a prompt reduction in serum dihydrotestosterone of >90% (by 2 weeks), which was maintained for the duration of the study, a decrease in prostate volume of 25.7% at two years, an improvement in Q_MAX _and a reduction in the risks of AUR (by 57%) and the need for BPH-related surgery (by 48%).

Though pain is rarely reported in connection with BPH, it is a feature of prostatitis, which is also common in older men [5] and can often be confused with BPH in the older male population [6]. In a study comparing men with prostatitis and BPH, pain during urination was a feature for 54% and 29% of the groups respectively [7].

The increasing recognition of the importance of patient-reported outcomes (PRO) in recent years has led to the development of a large number of PRO questionnaires. Within the treatment of BPH, this has included measures such as the Boyarsky Score [8], the International Prostate Symptom Score (IPSS) [9] and the BPH Impact Index (BII) [10], which have become accepted standard measures in the field.

Patient satisfaction with treatment, which includes patients' evaluations of the process and outcome of their treatment experience, is increasingly being evaluated in clinical trials and disease-management programs [11,12]. However, there are few reports about treatment satisfaction amongst BPH patients. Treatment satisfaction measures with evidence of reliability and validity are needed to evaluate BPH therapies in clinical studies to ensure that results are valid and meaningful to clinical practice. The objective of this study was to evaluate the validity and reliability of a new questionnaire developed to assess BPH-patient satisfaction with study medication.

## Methods

### The Clinical Trial

The Patient Perception of Study Medication (PPSM) was administered during a large multi-national clinical trial called CombAT (Combination of Avodart and Tamsolusin). This study was conducted in accordance with 'good clinical practice' (GCP) and all applicable regulatory requirements, including, where applicable, the 1996 version of the Declaration of Helsinki. Schulman Associates IRB approved the CombAT study on September 24, 2003, reference number 03-4400-0.

Men aged 50 years or over with a clinical diagnosis of BPH and an IPSS score of 12 or more points at screening were invited to participate in this four-year multi-centre, randomised, double-blind parallel-group study to assess whether combination therapy with dutasteride and tamsulosin is more effective than either monotherapy alone for improvement of symptoms and clinical outcomes. Prior clinical trials have demonstrated a treatment impact of dutasteride monotherapy on reducing symptoms within 3–6 months of starting treatment [1,4] and of alpha-1 adrenoreceptor antagonists such as tamsulosin monotherapy on reducing symptoms within one month of starting treatment [13].

Case definition included prostate volume ≥ 30 cc (by transrectal ultrasonography, TRUS), total serum Prostate Specific Antigen (PSA) ≥ 1.5 ng/mL at screening, maximum flow rate (Qmax) >5 mL/sec and minimum voided volume ≥ 125 mL at screening. Exclusion criteria included total serum PSA >10.0 ng/mL at screening; history or evidence of prostate cancer; previous prostatic surgery; history of flexible/rigid cyctoscopy or other instrumentation of the urethra within 7 days prior to screening; history of AUR within 3 months prior to screening; post-void residual volume >250 mL (suprapubic ultrasound) at screening; use of any 5-alpha-reductase inhibitor (e.g. Proscar, Propecia), any drugs with antiandrogenic properties (e.g. spironolactone, flutamide, bicalutamide, cimetidine, ketoconazole, progestational agents), or other drugs noted for gynaecomastia effects, or that could affect prostate volume, within past 6 months of the historical TRUS or screening vsit and throughout the study (other than as study medication). Subjects with a screening IPSS score of <12 (based on the first 7 items) were excluded from the study to ensure that only patients with moderate to severe LUTS were included.

The trial was conducted in Argentina, Belgium, Brazil, Bulgaria, Canada, Czech Republic, Denmark, Estonia, Finland, France, Germany, Greece, Hungary, Israel, Italy, Korea, Lithuania, Mexico, Netherlands, Norway, Philippines, Poland, Portugal, Puerto Rico, Romania, Russian Federation, Slovakia, South Africa, Spain, Taiwan, Thailand, Turkey, United Kingdom and the United States. The current study focuses on data obtained during the first two years of the study in the US amongst English-speaking patients only, using the original US English measure. Patients completed the PRO measures at baseline (after completion of the placebo run-in period) and at every 13-week clinic visit thereafter for the duration of the study period.

### Patient Report Outcome Measures

#### PPSM

At the time of study conception, there were only global satisfaction assessments available in which to assess satisfaction with BPH therapy. The PPSM (Appendix 1) was developed by GlaxoSmithKline (GSK) for use in this clinical trial to determine whether questions addressing satisfaction with individual symptoms provided additional useful information on patient satisfaction with BPH pharmacotherapy above and beyond what was already provided by global satisfaction. A draft of the questionnaire was developed on the basis of input from patient focus groups. This draft questionnaire was further refined by clinician input based on the objectives of the existing clinical trial. It is a 12-item questionnaire designed to quantify patients' satisfaction with the effect of the study treatment by focussing on specific changes experienced by patients during the study period in 4 areas – control of urinary symptoms (2 items), strength of urinary stream (2 items), 2 aspects of pain of urination (2 items each), effect on usual activities (2 items), with a single item asking about overall satisfaction. There is also a final item asking about whether the respondent would ask their doctor for this medication.

Each of the areas of interest includes an item asking about the patient's perception of how that aspect of the condition has changed since they began taking the study medication, set against a 7-point Likert-type response scale ranging from much improved to much worse and another item asks how satisfied the patient is with the effect of the study medication on that aspect of their condition, set against a 7-point Likert-type response scale ranging from very satisfied to very dissatisfied. The area addressing pain looks at 2 aspects of pain – pain prior to urination and pain during urination. The overall satisfaction item has the same response scale as the other satisfaction items. The final item, question 12, is set against a discrete 3-point scale – 'yes', 'no' or 'not sure'. The PPSM yields scores for each area of interest in addition to a total score (items 1–11), which is calculated simply by adding each individual raw score. The control of urinary symptoms, strength of urinary stream and effect on usual activities scores range from 2 to 14; the pain scores range from 0 to 28; the overall satisfaction score ranges from 1 to 7. The total score for items 1–11 ranges from 7 to 77. Higher scores indicate lower satisfaction. Patients completed the PPSM at baseline (after completion of the placebo run-in period) and at every 13-week clinic visit thereafter during the study treatment period.

#### IPSS

The IPSS [9] is a 7-item urinary symptom scale, each with a 6-point frequency response scale. (Items 1 to 6: 0 = Never, 1 = About 1 time in 5, 2 = About 1 time in 3, 3 = About 1 time in 2, 4 = About 2 times in 3, 5 = almost always). Item 7 asks how many times in the past month the respondent has to get up in the night to urinate (response scale: 0 = none, 1 = 1 time, 2 = 2 times, 3 = 3 times, 4 = 4 times, 5 = 5 or more times). There is also an independent eighth item which addresses overall quality of life – "If you were to spend the rest of your life with your urinary condition just the way it is now, how would you feel about that?" – against a 7-point Likert-type response scale (0 = delighted, 1 = pleased, 2 = mostly satisfied, 3 = mixed, 4 = mostly dissatisfied, 5 = unhappy, 6 = terrible). The IPSS yields a total score for the 7 symptom items, ranging from 0 to 35, with higher scores indicating greater symptom severity. The Quality of Life (QoL) item scores range from 0 to 6, with higher scores indicating poorer quality of life. Validity of the IPSS has previously been widely demonstrated [e.g. [14,15]]. Patients completed the IPSS at baseline (after completion of the placebo run-in period) and at every 13-week clinic visit thereafter during the study treatment period.

#### BII

The BII [10] is a 4-item instrument which assesses the overall impact of BPH on patients' general well-being. It measures aspects of physical discomfort, worry, bother, and interference with everyday activities. The items about physical discomfort have a 4-point Likert-type response scale (0 = none, 1 = only a little, 2 = some, 3 = a lot); the bothersomeness item also has a 4-point response scale (0 = not at all bothersome, 1 = bothers me a little, 2 = bothers me some, 3 = bothers me a lot); the interference with everyday activities item has a 5-point Likert-type response scale (0 = none of the time, 1 = a little of the time, 2 = some of the time, 3 = most of the time, 4 = all of the time). The BII yields a total score for all 4 items, ranging from 0 to 13, with higher scores indicating a greater impact on patients' general well-being. Validity of the BII has been previously demonstrated (e.g. [10]). Patients completed the BII at baseline (after completion of the placebo run-in period) and at every 13-week clinic visit thereafter during the study treatment period.

### Evaluation of PPSM psychometric properties

Psychometric testing of the PPSM was conducted using standardized procedures [16] and instrument review criteria developed by the Scientific Advisory Committee of the Medical Outcomes Trust [17], including: item characteristics, factor structure, reliability, validity and responsiveness. All psychometric analyses were based on data collected at one year (visit 6), with the exception of assessments of responsiveness in the clinical trial that also used data from baseline and other follow-up visits (visits 2, 3 and 10 – baseline, 13 weeks and 2 years respectively). All 'not applicable' responses were coded as 'missing' and were therefore not included as responses in the analyses. Psychometric assessments were made using SPSS (Statistical Package for the Social Sciences) for Windows version 15.0.

#### Item characteristics

Mean scores, score ranges, missing data (items with >5% missing), ceiling effects (>50% indicating 'much improved' or 'very satisfied' on any item) and floor effects (>50% indicating 'much worse' or 'very dissatisfied' on any item) were examined for each PPSM item.

#### Factor structure

Exploratory factor analyses procedures were performed on the correlation matrices derived from the items comprising the questionnaire. Principal component analysis with rotational methods (Varimax) were employed to achieve a meaningful set of factors. The appropriate number of factors to be extracted was determined as a function of the proportion of common variance accounted for, residuals analysis and scree plot examination, along with clinical and theoretical interpretability. The un-weighted scales were comprised of those items with factor loadings of at least 0.30.

#### Reliability

Cronbach's alpha was calculated using the one-year (visit 6) data to assess internal consistency, or the degree of association between the item and scale scores [18]. Reproducibility (test/retest reliability) could not be assessed due to the clinical trial design. Cronbach's alpha values of at least 0.70 are considered desirable for performing group-level comparisons [17,18].

#### Validity

Convergent validity, a type of construct validity, involves comparing a PRO measure of one concept to another logically-related measure with the same concept. If previous predictions of association are accurate, then convergent validity is achieved. Convergent validity for the PPSM was assessed by using Pearson's correlation to measure the association between the total and subscale scores of the PPSM measure and the IPSS.

Known-groups validity involves assessing whether or not a PRO is able to distinguish between two or more recognized groups with theoretically different levels of the outcome to be measured. In this analysis, the PPSM was assessed using definitions of BPH-related severity in the IPSS (mild, moderate, severe) and BPH-related impact in the BII (low, medium and high). Known-groups validity was also explored for the different treatment arms – combination (dutasteride and tamsulosin), dutasteride and placebo, and tamsulosin and placebo.

#### Responsiveness

Responsiveness is the ability of an instrument to detect small but important changes [19,20]. Change scores were calculated using the difference between baseline (visit 2) and 3 of the follow-up visits – 13-week follow-up (visit 3), one-year follow-up (visit 6) and two-year follow-up (visit 10). These were interpreted according to expected treatment effects.

## Results

### Sample characteristics at baseline

Data was obtained from a total of 879 patients, ranging in age from 49 to 86 years (mean age 66.7 years). Of these, 47% had previously taken an alpha blocker and 14% had previously been treated with a 5ARI.

IPSS and BII scores were included only for those patients who also provided PPSM scores and not all patients provided PPSM data at baseline. Baseline IPSS scores ranged from 1 to 35, with a mean of 16.85. Baseline BII scores ranged from 0 to 13, with a mean of 4.72. In response to the IPSS QoL item, " If you were to spend the rest of your life with your urinary condition just the way it is now, how would you feel about that?", patients variously reported being 'pleased' (2.6%), 'mostly satisfied' (11.7%), 'mixed' (34.2%), 'mostly dissatisfied' (27.9%), 'unhappy' (17%) and 'terrible' (6.7%).

With no independent measure of pain included, the number of patients responding 'not applicable' to the PPSM pain items (questions 5 and 7) were taken as an indicator of the numbers of patients who did not experience pain. These were found to be 31.1% and 32.3% respectively.

### Measurement Structure of the PPSM

The exploratory factor analysis of items 1–11 suggested that the items loaded onto 2 factors – items 1–4 and 9–11 loading onto one factor (PPSM-Global) and all the pain items loading onto another (PPSM-Pain). The Global item loadings ranged from .75 to .90 (cumulative percent of variation was 68%) and the Pain item loadings ranged from .87 to .91, with the cumulative percent of variation at 83%. Therefore the final scoring algorithm for the 12 items consisted of 1) the Global score (items 1–4 and 9–11), 2) the Pain score (items 5–8), 3) Total Score (items 1–11) and 4) Item 12 about whether the patient would ask their doctor for the medication.

### PPSM Item characteristics

Looking at individual items, based on the criteria of >5%, we found that there was no problem with missing data. There were no ceiling or floor effects (all of the items had <16% responding at one extreme or the other). Item-to-item correlations were all above the .70 level and all of them were significant at the 0.01 level. Item-to-total score correlations ranged from .76 to .92 (all significant at the 0.01 level). Item-to-total correlations for PPSM-Global ranged from .76 to .92 (all significant at the 0.01 level) and item-to-total for PPSM-Pain ranged from .90 to .92 (all significant at the 0.01 level). All correlations were corrected for item overlap.

### Reliability

The PPSM showed high internal consistency, greater than the desirable .70 – the PPSM Total Score had an *alpha *score of .97, PPSM-Global had an *alpha *score of .95 and PPSM-Pain had an *alpha *score of .96 (see Table 1). The other areas of interest included within the measure (control of urinary symptoms, strength of urinary stream and effect on usual activities) also showed good internal consistency (*alpha *ranged from .88 to .89).

**Table 1:** Alpha Statistics on the PPSM at 1 Year

***PPSM Questionnaire***	**Alpha coefficients**
PPSM Total Score	.97

PPSM-Global	.95

PPSM-Pain	.96

### Validity

Convergent validity of the PPSM scores ranged from .48 to .58 for the IPSS and .31 to .45 for the BII. All were significant at the 0.01 level (see Table 2).

**Table 2:** Convergent Validity of the PPSM at 1 Year

***PPSM Questionnaire***	**IPSS**	**BPH Impact Index**
PPSM Total Score	.58**	.45**

PPSM-Global	.58**	.42**

PPSM-Pain	.48**	.31**

** Correlation is significant at the 0.01 level (2-tailed)

Known-groups validity scores according to BPH severity as indicated by IPSS and BII categories are presented in Table 3. PPSM Total Scores, PPSM-Global and PPSM-Pain were all significantly different for patients who were classed as mild, moderate or severe on the IPSS. The scores were also significantly different for each point of the IPSS QoL score and for the low medium and high impact categories on the BII. The F statistics for the PPSM Total Score, PPSM-Global and PPSM-Pain comparisons with the IPSS, IPSS QoL Item and BII were 31.25, 13.57 and 16.56, 52.53, 42.39 and 26.97, and 17.34, 6.97 and 6.96 respectively. All differences were significant at the 0.001 level.

**Table 3:** Known-Groups Validity Statistics for the PPSM at 1 Year

	***PPSM Total Score***			***PPSM-Global***			***PPSM-Pain***		
***Health Outcome Measures and Ranges***	**N**	**F-Statistic**	**Mean (SD)**	**N**	**F-Statistic**	**Mean (SD)**	**N**	**F-Statistic**	**Mean (SD)**

**IPSS^+^**									
									
Level 1	25	31.25***	21.44 (9.89)	65	52.53***	15.21 (5.83)	25	17.34***	8.16 (4.79)
Level 2	84		30.22 (9.31)	139		20.09 (6.05)	85		10.95 (3.99)
Level 3	21		43.57 (9.76)	40		28.17 (7.67)	21		15.19 (3.24)

**IPSS HRQoL^++^**									
									
Level 1	27	13.57***	21.51 (9.88)	43	42.39***	13.16 (5.20)	27	6.97***	8.29 (5.02)
Level 2	39		27.33 (9.95)	84		17.40 (5.49)	40		10.07 (4.27)
Level 3	43		33.65 (7.95)	73		21.91 (4.87)	43		11.95 (3.44)
Level 4	15		39.00 (10.82)	32		26.87 (7.59)	15		13.73 (3.69)
Level 5	9		41.55 (14.18)	17		28.88 (8.10)	9		14.33 (4.63)

**BII^+++^**									
									
Level 1	93	16.56***	27.25 (10.40)	188	26.97***	18.26 (6.59)	94	6.96***	10.05 (4.47)
Level 2	36		36.58 (9.86)	57		24.82 (7.37)	36		12.88 (3.77)
Level 3	5		46.00 (11.46)	6		30.00 (7.46)	5		14.20 (5.84)

* p < 0.05, ** p < 0.01, *** p < 0.001

^+ ^Level 1 – mild (scores of 0–7); Level 2 – moderate (scores of 8–19); Level 3 – severe (scores of 20–35)

^++^Level 1 – *Delighted/Pleased*; Level 2 – *Mostly satisfied*; Level 3 – *Mixed*; Level 4 – *Mostly dissatisfied*; Level 5 – *Unhappy/Terrible*

^+++^Level 1 – low impact (scores of 0–4); Level 2 – medium impact (scores of 5–8); Level 3 – high impact (scores of 9–13)

### Treatment effects

PPSM-Global scores by treatment arm for baseline and at 2 years are presented in Table 4. PPSM scores at baseline for the combination therapy, dutasteride and tamsulosin treatment groups were 25.55, 25.40 and 25.66 respectively. At 2 years (visit 10), the scores were 17.76, 20.32 and 20.47 respectively.

**Table 4:** Sensitivity to change: PPSM-Global by Study Treatment Arms at 2 Years

		***PPSM Questionnaire – PPSM-Global***			
		**Visit 2** **(Baseline)**	**Visit 10** **(2 Years)**	**Change:** **Visit 2 to Visit 10**	**Effect Size**

**Treatment Arm**	**N**	**Mean** **(SD)**	**Mean** **(SD)**	**Mean** **(SD)**	

0.5 mg dutasteride + 0.4 mg tamsulosin	158	25.55 (6.61)	17.76 (6.88)	-7.79 (7.21)	-1.17

0.5 mg dutasteride + placebo	190	25.40 (5.20)	20.32 (7.57)	-5.07 (8.08)	-0.97

0.4 mg tamsulosin + placebo	161	25.66 (6.09)	20.47 (7.55)	-5.19 (7.88)	-0.85

Notes: Sample for separate visit scores includes everyone that has a PPSMQ Total Score (excl. pain) at both time points.

Effect size: Mean change score (visit 2 to visit 10) divided by standard deviation of visit 2 score

## Discussion

Measuring satisfaction with medication provides important outcome information from the patient's perspective as to their experience with the therapy and their willingness to ask their physician for the treatment. Psychometrically-sound instruments that include relevant items and or domains are necessary for assessing treatment satisfaction. This study demonstrates the reliability and validity of the PPSM as a measure of patient satisfaction with BPH treatment. It was also responsive to changes in symptom severity and treatment differences. Based on the study findings, the PPSM is an acceptable measure for assessing satisfaction with medication in future clinical studies of BPH medications.

For instruments to be responsive to change, the items contained in the questionnaire should have few missing items and minimal floor and ceiling effects. In terms of missing data, none of the items had greater than 5% unanswered, indicating patients had no problems understanding and responding to each item. Similarly none of the items showed ceiling or floor effects, indicating an appropriate range of response choices. All items were highly correlated with the PPSM Total Score, the PPSM-Global and the PPSM-Pain.

Item-to-item correlations were all above the 0.70 level, and several items were correlated with a number of other items, which would indicate that some of the items might be redundant. However, items 2, 3, 4, 11 and 12 were identified by patients as distinct and measuring different constructs during the development work. Items 1, 5, 7 and 9 were derived from a single item ('Since you began talking the study medication, how have your urinary problems changed?') to address specific aspects of 'urinary symptoms', which patients also identified as being distinct from other items. Finally, items 6, 8 and 10, all asking about patient satisfaction relating to changes in specific symptoms, were felt to balance the symptom change items and had good face-validity. Thus it was not felt necessary to exclude any items due to redundancy or overlap in information captured.

As the exploratory factor analysis indicated that the items loaded onto two factors – PPSM-Global and PPSM-Pain, subsequent analyses were carried out on the PPSM Total Score, PPSM-Global and PPSM-Pain.

Reliability, using Cronbach's alpha, was confirmed for the Total Score, PPSM-Global and PPSM-Pain, where high internal consistency was demonstrated with alpha values above 0.90. Test-retest reliability could not be measured because of the clinical trial design – the measurement points being 13 weeks apart. Not being able to assess test-retest reliability for the PPSM may not be of great concern, as satisfaction is typically assessed at a single point in time (e.g. the end of study or the end of treatment).

The convergent validity of the PPSM was good, demonstrating that although scores on the PPSM are correlated with the scores on the IPSS and BII, it is also measuring some different constructs. Known-groups validity was demonstrated using classifications based on the BPH severity of the patients as measured by the IPSS and the BII. The PPSM Total Score, PPSM-Global and PPSM-Pain were all able to discriminate between levels of severity, with satisfaction shown to be higher for patients with lower symptom severity on the IPSS and lower impact on the BII. Similarly, patients reporting lower quality of life on the IPSS QOL item reported lower satisfaction on the PPSM.

At baseline (visit 2), there was no apparent difference in satisfaction between the treatment groups. Tamsolusin has a fast onset of action, and this is seen in the higher satisfaction scores for both tamsulosin monotherapy and combination therapy by visit 3 (13 weeks). The patient-perceived changes in symptoms while taking dutasteride has been reported to occur as early as 3–6 months. The satisfaction scores for dutasteride are approaching those of tamsulosin monotherapy at year 1 and are superior to tamsulosin by year two. By visit 10 (2 years), the combination treatment group reported greater satisfaction than the other treatment groups.

### Recommendations for use

Due to low prevalence of pain in BPH patients and the psychometric performance of the PPSM with regards to the pain items, it is recommended that the PPSM-Global items be used alone, resulting in an 8-item measure. The total score for this 8-item measure is obtained by adding all the raw scores for items 1–7, which gives a score range of 7 to 49. Responses to item 8, "Would you ask your doctor for the medication you received in this study?" are to be used independently.

Given the strength of the psychometric performance of the pain component, however, it is suggested that the entire 12-item measure can be used in populations for which pain is a feature of their condition. In this case, in addition to the score obtained for items 1–8 as outlined above, the pain component can be scored by adding the raw scores for these items, which gives a pain score range of 0–28.

### Further development of the PPSM

Exploration of the PPSM as a uni-dimensional measure of patient satisfaction may be useful. The current version of the measure includes both change and satisfaction with change items, and whilst all of these (excluding the pain items) clearly load onto a single factor, given the high level of item-to-item correlations, it is recommended that future work focuses on the analysis of data elicited only by those items which specifically address patient satisfaction (items 2, 4, 6, 8, 10, 11 & 12).

### Study Limitations

Several limitations should be considered when interpreting these psychometric results. Generalizability of findings may be limited by characteristics of the study population – including entry criteria such as requiring a patient to have a minimum score of 12 or greater in the IPSS and a PV greater than 30 cc. Further studies are needed to examine the psychometric characteristics of the PPSM in a broader and more representative sample of BPH patients. These studies should explore the possibility of item redundancy and the minimally important difference (MID) for patients with varying levels of symptom severity at baseline and between treatment groups. Finally, similar analyses of the data elicited in other countries would be desirable to assess the extent of cultural variation on questionnaire performance which would facilitate the decisions on how the questionnaire might be used in multi-national clinical studies.

## Conclusion

The PPSM is a disease-specific patient-reported outcome measure designed to evaluate patient satisfaction with treatment for BPH by evaluating patient perceptions of change and their satisfaction with that change. Using data from a randomized clinical trial in the USA, the results support the reliability, validity and responsiveness to change of the PPSM. Its performance in these analyses, and its emphasis on satisfaction, suggest that it might be an important addition to the existing outcome measures which are used to assess BPH symptoms and their treatment.

## Competing interests

The authors declare that they have no competing interests.

## Authors' contributions

LB participated in the study design, the analysis and interpretation of data and revision of the manuscript. AG participated in the study design, analysis and interpretation of data and drafting the manuscript. BM participated in the acquisition of data and the revision of the manuscript. All authors read and approved the final manuscript.

## Appendices

### Appendix 1: Patient Perception of Study Medication: Satisfaction

Item numbers for the PPSM-Global items are found in brackets where they are different.

Q1. Since you began taking the study medication, how has control of your urinary problems changed?

Q3. Since you began taking the study medication, how has the strength of your urinary stream changed?

Q9(5). Since you began taking the study medication, how has the way your urinary problems interfere with your ability to go about your usual activities changed?

Response Scale: Much improved; Improved; Somewhat improved; No change; Somewhat worse; Worse; Much worse/much less control

Q2. How satisfied are you with the effect of the study medication on control of you urinary problems?

Q4. How satisfied are you with the effect of the study medication on the strength of your urinary stream?

Q6(-). How satisfied are you with the effect the study medication has on your pain prior to urinating?

Q8(-). How satisfied are you with the effect the study medication has on your pain during urination?

Q10(6). How satisfied are you with the effect the study medication has on your ability to go about your usual activities without interference from your urinary problems?

Q11(7). Overall, how satisfied are you with the study medication and its effects on your urinary problems?

Response scale: Very satisfied; Satisfied; Somewhat satisfied; Neutral (neither satisfied nor dissatisfied); Somewhat dissatisfied; Dissatisfied; Very dissatisfied

Q5(-). Since you began taking the study medication, how has your pain prior to urinating changed?

Q7(-). Since you began taking the study medication, how has your pain during urination changed?

Response scale: Much improved/much less pain; Improved; Somewhat improved; No change; Somewhat worse; Worse; Much worse/much more pain; Not applicable

Q12(8). Would you ask your doctor for the medication you received in this study?

Response scale: Yes; No; Not sure
